# Granulomatous inflammation in cartilage-hair hypoplasia: risks and benefits of anti-TNF alpha monoclonal antibodies

**DOI:** 10.1186/1546-0096-9-S1-P39

**Published:** 2011-09-14

**Authors:** D Moshous, I Meyts, S Fraitag, C Janssen, M Debré, F Suarez, J Toelen, K De Boeck, T Roskams, A Deschildre, C Picard, C Bodemer, C Wouters, A Fischer

**Affiliations:** 1 Department of Pediatric Immunology and Hematology, Assistance Publique-Hôpitaux de Paris, Hôpital NeckerEnfants-Malades, Paris, France; 2 Université Paris Descartes, Faculté de Médecine de l'Université RenéDescartes, Institut Fédératif de Recherche Necker Enfants-Malades (IFR94), Paris, France; 3 Institut National dela Santé et de la Recherche Médicale, Unité U768, Laboratoire du Développement Normal et Pathologique du Système Immunitaire, Paris, France; 4 Pediatric Immunology and Rheumatology, University Hospital Gasthuisberg, Leuven, Belgium; 5 Department of Pathology, Assistance Publique-Hôpitaux de Paris, Hôpital Necker-Enfants Malades, Paris, France; 6 Department of Pathology, University Hospital Gasthuisberg, Leuven,Belgium; 7 Department of Hematology, Assistance Publique-Hôpitaux de Paris, Hôpital Necker Enfants-Malades, Paris, France; 8 Unité de pneumologie pédiatrique, Hôpital Jeanne de Flandre, Centre Hospitalier Régional, Universitaire, Lille, France; 9 Centre d’Etude des Déficits Immunitaires, Assistance Publique-Hôpitaux de Paris, Hôpital Necker-Enfants Malades, Paris, France; 10 Department of Dermatology, Assistance Publique-Hôpitaux de Paris, Hôpital Necker-Enfants Malades, Paris, France

## Background

Cartilage-hair hypoplasia (CHH) is a rare autosomal recessive disorder characterized by short-limbed skeletal dysplasia. Some patients also develop defects in cell-mediated immunity and antibody production. Granulomatous inflammation has been described in patients with various forms of primary immunodeficiencies but, to date, has not been reported in patients with Cartilage-hair hypoplasia.

## Aims

To describe granulomatous inflammation as a novel feature in patients with CHH, assess associated immunodeficiency and evaluate treatment options.

## Methods

In a retrospective, observational study, we collected clinical data on 21 patients with CHH in order to identify and further characterize individuals with granulomatous inflammation.

## Results

Four unrelated patients with CHH (with variable degrees of combined immunodeficiency) developed epithelioid cell granulomatous inflammation in the skin and visceral organs. Anti Tumor necrosis factor alpha monoclonal antibody therapy in 3 of these patients led to significant regression of granulomas. However, one treated patient developed fatal progressive multifocal leukoencephalopathy due to the JC polyomavirus. In two patients, immune reconstitution after allogeneic hematopoietic stem cell transplantation led to the complete disappearance of granulomas.

## Conclusion

To the best of our knowledge, this is the first report of granulomatous inflammation in patients with CHH. Although Tumor necrosis factor alpha antagonists may effectively suppress granulomas, the risk of severe infectious complications limits their use in immunodeficient patients.

